# Endogenous retroelement expression in the gut microenvironment of people living with HIV-1

**DOI:** 10.1016/j.ebiom.2024.105133

**Published:** 2024-04-26

**Authors:** Nicholas Dopkins, Tongyi Fei, Stephanie Michael, Nicholas Liotta, Kejun Guo, Kaylee L. Mickens, Brad S. Barrett, Matthew L. Bendall, Stephanie M. Dillon, Cara C. Wilson, Mario L. Santiago, Douglas F. Nixon

**Affiliations:** a Division of Infectious Diseases, Department of Medicine, Weill Cornell Medicine, New York, NY, USA; b Department of Medicine, University of Colorado School of Medicine, Aurora, CO, USA; c RNA Bioscience Initiative, University of Colorado School of Medicine, Aurora, CO, USA; d Department of Immunology and Microbiology, University of Colorado School of Medicine, Aurora, CO, USA

**Keywords:** Human endogenous retrovirus (HERV), Interferons, Human immunodeficiency virus type I (HIV-I), Endogenous retroelement (ERE), Long interspersed nuclear element (LINE)

## Abstract

**Background:**

Endogenous retroelements (EREs), including human endogenous retroviruses (HERVs) and long interspersed nuclear elements (LINEs), comprise almost half of the human genome. Our previous studies of the interferome in the gut suggest potential mechanisms regarding how IFNb may drive HIV-1 gut pathogenesis. As ERE activity is suggested to partake in type 1 immune responses and is incredibly sensitive to viral infections, we sought to elucidate underlying interactions between ERE expression and gut dynamics in people living with HIV-1 (PLWH).

**Methods:**

ERE expression profiles from bulk RNA sequencing of colon biopsies and PBMC were compared between a cohort of PLWH not on antiretroviral therapy (ART) and uninfected controls.

**Findings:**

59 EREs were differentially expressed in the colon of PLWH when compared to uninfected controls (padj <0.05 and FC ≤ −1 or ≥ 1) [Wald's Test]. Of these 59, 12 EREs were downregulated in PLWH and 47 were upregulated. Colon expression of the ERE loci LTR19_12p13.31 and L1FLnI_1q23.1s showed significant correlations with certain gut immune cell subset frequencies in the colon. Furthermore L1FLnI_1q23.1s showed a significant upregulation in peripheral blood mononuclear cells (PBMCs) of PLWH when compared to uninfected controls suggesting a common mechanism of differential ERE expression in the colon and PBMC.

**Interpretation:**

ERE activity has been largely understudied in genomic characterizations of human pathologies. We show that the activity of certain EREs in the colon of PLWH is deregulated, supporting our hypotheses that their underlying activity could function as (bio)markers and potential mediators of pathogenesis in HIV-1 reservoirs.

**Funding:**

US 10.13039/100000002NIH grants 10.13039/100000054NCI CA260691 (DFN) and 10.13039/100000060NIAID UM1AI164559 (DFN).

**Research in context**

**Evidence before this study**

Previous studies have demonstrated that HIV-1 infection alters the physiology of gastrointestinal (GI) tissues, where it forms quiescent reservoirs. In chronic HIV-1, the sustainment of antiviral immune responses through poorly understood mechanisms likely contributes to pathogenesis and prohibits effective clearance of the viral reservoirs. EREs comprise a substantial portion of the human genome that has been implicated in HIV-1 pathogenesis but remains understudied in comparison to coding genes.

**Added value of this study**

Colon biopsies from PLWH demonstrate differential expression of 59 EREs when compared to uninfected controls. Further analysis into cellular composition of the gut microenvironment and plasma viral load demonstrate that the EREs LTR19_12p13.31 and L1FLnI_1q23.1s may contribute to pathogenesis and function as biomarkers of gut-specific HIV-1 pathologies.

**Implications of all the available evidence**

High expression of LTR19_12p13.31 and L1FLnI_1q23.1s can function as markers of GI tract HIV-1 reservoirs, as well as potentially possess undefined roles in human health.

## Introduction

Human immunodeficiency virus type I (HIV-1) is a retrovirus that infects immune cells by binding to receptors on the cell surface.^1^ HIV-1 infects host cells by integrating a copy of the viral genome into the host genome, resulting in proviruses that then hijack the host cell's replicative machinery to produce the building blocks of infectious particles.^1^ The HIV-1 lifecycle may rapidly deplete host CD4+ T cell populations and disrupt global immune dynamics.^2^ Disruptions to the host immune system predispose people living with HIV-1 (PLWH) to life-threatening comorbidities.^3^ Since 1996, life expectancy and quality of life for PLWH have risen due to the advent of antiretroviral therapies (ARTs) that pharmacologically inhibit key processes of the retroviral life cycle.^4^ Unfortunately, ART merely suppresses retroviral processes, emphasizing the need for cure strategies that rid individuals of latent proviral reservoirs which may resume infectious particle formation after a lapse in treamtent.^5^ One such reservoir site is the gastrointestinal (GI) associated lymphoid tissue (GALT),^6^ which is particularly rich in CD4+ T cells.^7^ HIV-1 induced immunopathologies in the GALT are associated with severe comorbidities in PLWH.^8^^,^^9^ Many of these pathologies could be sustained by the accumulation of plasmacytoid dendritic cells (pDCs) that are trafficked to the GI tract during chronic HIV-1 infection.^10^ Models of Simian Immunodeficiency Virus (SIV), a retrovirus closely related to HIV-1, demonstrate that pDCs traffic from the circulation, where they are depleted, to the GALT, where their overabundance contributes to inflammation through the release of cytokines.^11^, ^12^, ^13^ In the acute stages of HIV-1 infection, pDCs produce type 1 interferons (IFN-Is) as one of the first lines of defense against the virus,^14^ and are therefore protective. However, sustained IFN-I production during the chronic stages of infection exhausts and deregulates antiviral immunity, negatively impacting the health of PLWH.^10^^,^^14^, ^15^, ^16^

To better characterize how immunopathologies in PLWH develop, we investigated how endogenous retroelement (ERE) expression may underlie the development of HIV-1 induced immune deregulation. EREs are highly abundant sequences estimated to make up roughly ∼40% of the human genome.^17^ In humans, EREs include long interspersed nuclear elements (LINEs), short interspersed nuclear elements (SINEs), and human endogenous retroviruses (HERVs).^17^ Previous studies have demonstrated that ERE activity is highly susceptible to change during infection with HIV-1.^18^, ^19^, ^20^ While IFN-I signaling may activate EREs proximal to interferon stimulated genes (ISGs), other EREs, such as HERV elements, can be directly trans-activated by HIV-1 cofactors due to high sequence homology between endogenous and exogenous retroviruses.^20^^,^^21^ This homology can be so substantial that certain HERV-K products can be incorporated in the assembly of HIV-1 particles.^22^, ^23^, ^24^ ERE activity also functions as a major regulator of transcriptional networks that have been co-opted in organismal physiology.^25^ In immunity, examples of ERE activity cooption suggest their intimate association with the host mounted responses.^26^, ^27^, ^28^ Recurrent observations continually reinforce hypotheses that EREs likely participate in human health and disease through poorly understood mechanisms.^29^ As ERE activity is altered during HIV-1 infection, these changes may then impact host gene expression,^30^ packaging of HIV-1 particles,^22^, ^23^, ^24^ and immunity.^31^^,^^32^ For the purpose of further defining HIV-1 mediated gut pathogenesis, we sought to characterize how ERE activity at the RNA level is modulated in the gut microenvironment of PLWH. Since EREs have been largely overlooked in genomic approaches to classify underlying components of human pathologies, we hypothesize that their consideration would identify markers of HIV-1 pathogenesis in the gut.

ERE expression at the RNA level was quantified in colon biopsies collected from a cohort of viremic individuals who are not on ART^15^ to identify those best correlating with viral load. We next overlaid these findings with IFN-I stimulated gut CD4+ T cells to estimate if the differential expression of EREs in the gut of PLWH is driven by the HIV-1 lifecycle or by innate immunopathologies. Collectively, our analyses demonstrate that a subset of ERE loci are impacted in PLWH. The bulk of PLWH-upregulated EREs in the gut were not similarly upregulated by IFN-I stimulation of gut CD4+ T cells, suggesting their activation by other aspects of the HIV-1 life cycle or the host response. Two identified EREs, LTR19_12p13.31 and L1FLnI_1q23.1s, further correlated with myeloid and lymphoid immune cell abundances. Collectively, incorporation of the GI-specific expression of LTR19_12p13.31 and L1FLnI_1q23.1s can better define HIV-1 driven immunopathologies of the GALT, and the expression of these elements may possess underlying roles in pathogenesis.

## Methods

### Locus-specific quantification of ERE expression at the RNA level

RNA sequencing FASTQ files of colon pinch biopsies and interferome arrays of uninfected CD4+ T cells were obtained from a previously published study^15^ under the Bioproject accession numbers “PRJNA558500” and “PRJNA558974”, respectively. RNA sequencing FASTQ files performed on the PBMC fraction are available upon request and to be published under the Bioproject accession number “PRJNA659373”. FASTQ files were aligned to the human genome build 38 (hg38) using STAR (v2.7.9.a).^33^ STAR alignment was performed using the parameters “--outSAMstrandField intronMotif --outFilterMultimapNmax 200 --winAnchorMultimapNmax 200” for the purpose of capturing multimapping ERE reads. We then used Telescope (v1.0.3),^34^ which utilizes an expectation-maximization algorithm to improve upon the definition of ubiquitously mapping ERE transcripts to a custom annotation, for locus-specific approximation of ERE expression. The Telescope assign module was performed using the parameters “--theta_prior 200000 -- max_iter 1000” to align reads to a custom annotation of EREs accessible at https://github.com/mlbendall/telescope_annotation_db. Metadata for the ERE annotation predicting the intronic, exonic, and intergenic status of individual loci in the Telescope annotation relative to coding gene annotations can be found at https://github.com/liniguez/Telescope_MetaAnnotations. Original code produced for analysis can be accessed at https://github.com/NicholasDopkins/JuneHIVMucosa.

### Differential expression analysis

Lowly abundant EREs were filtered out from downstream analysis by ensuring that all EREs possessed at least 2 reads within 10% of the total samples. Differential expression analysis was performed between the ERE expression profiles of uninfected control and PLWH colon biopsies using standard DESEQ2 (v1.30.1)^35^ parameters. Differential expression analysis was performed between the ERE expression profiles of IFN-I stimulated gut CD4+ T cells using DESEQ2 with the parameters “parallel = T″ and “betaPrior = T”. Results were extracted as DESEQ objects, with a numbered contrast of each group compared against all others. For the purpose of identifying differentially expressed EREs, we determined any tested loci to possess a log2 fold change of >1 or < −1 to indicate biological significance and an adjusted p value (padj) of <0.05 [Wald's Test] to indicate statistical significance. All monitored loci that surpassed the log2 fold change threshold and possessed a padj value of <0.05 were then considered to be differentially expressed. Differentially expressed EREs were visualized with the packages pheatmap (v1.0.12), ggVennDiagram (v1.2.2)^36^ and EnhancedVolcano (v1.8.0).^37^ Individual EREs were visualized with a custom function that overlays geom_jitter and geom_boxplot functions provided by ggplot2 v (3.3.5)^38^ to produce a Tukey box and whisker plot that displays individual replicates. Clinical parameters of immunopathologies were provided by previously published metadata^15^^,^^16^^,^^39^^,^^40^ and a table summary is available at https://github.com/NicholasDopkins/JuneHIVMucosa/blob/main/Metadata.csv. Principal coordinate analyses (PCA) were performed on variance stabilizing transformed DESEQ outputs in order to visualize if untested confounding factors explained observations pertaining to differential expression analyses. PCA generation was performed using PCAtools (v2.2.0) with the variable parameter set to “removeVar = 0.1”.

### Integrative genomics viewer

National Center for Biotechnology Information (NCBI) Reference Sequence Database (Refseq),^41^ ENCyclopedia Of DNA Elements^42^ (Gencode), and Telescope^34^ annotations of the human genome were loaded into Integrative Genomics Viewer^43^ (IGV) for alignment of StringTie^44^ reconstructed transcriptomes from uninfected controls and PLWH samples.

### Statistics

The default Wald testing parameters within DESEQ2^35^ that provide a z score using shrunken estimates of log fold change divided by standard error and a Benjamini-Hochberg correction of p values were utilized for the identification of significantly deregulated EREs between uninfected controls and PLWH samples. Statistical values for correlations between clinical parameters, cytometric abundances, and ERE expression were conducted using R values were calculated using Spearman's correlation coefficient and are represented to a definition of 2 significant digits. Spearman's correlation coefficient values provided by ggplot2 with the shaded bands representing 95% confidence intervals (CIs) listed in the accompanying figure legends. Listed Spearman's correlation coefficient R value CIs were generated using the R Package RVAideMemoire (v0.9-83-7). Positive R values indicating a direct correlation are assumed as positive correlations and negative values indicating indirect correlations are assumed as negative correlations. To elucidate values of significance regarding of ERE expression between experimental groups, t testing, t testing with Bonferroni adjustment of p values for multiple comparisons, and one-way ANOVA testing were used to gather significance values when stated. The equality of variances between 2 groups was performed using an F-test prior to unpaired t testing. The equality of variances between greater than 2 groups was performed using Bartlett's test prior to ANOVA testing. Variance testing results are listed in the figure legends when applicable. Mann–Whitney U testing and Fisher's exact testing were conducted on parameters of the clinical cohort. P values are represented to a definition of 2 significant digits. For data interpretation, all p values < 0.05 are referred to as significant.

### Clinical cohort

ERE expression analyses were conducted using FASTQ files from previously generated RNAseq data collected from colon tissues and PBMCs collected from 19 PLWH and 13 age- and sex-matched controls, and associations with features of HIV-1 pathogenesis were determined using archived datasets of colon and systemic immunological and virological parameters.^15^^,^^16^^,^^39^^,^^40^ On average, PLWH had been infected with HIV-1 (defined by the first HIV-1 seropositive test) for 5.25 ± 4.8 (mean ± SD) years. PLWH were ART-naïve or were not on ART for more than 7 days in the preceding 6 months, and had CD4 T cell counts >200 cells/μl within 3 months of clinical visit. Exclusion criteria for both cohorts are extensively detailed elsewhere.^39^ In addition to blood samples, study participants underwent a flexible sigmoidoscopy with multiple colon pinch biopsies obtained. Clinical characteristics for the study participants included in this current study are detailed in Supplemental Table S1.

### Immune system characterization of PLWH and uninfected controls

Methods used to generate the archived datasets have been extensively detailed.^15^^,^^16^^,^^39^^,^^40^^,^^45^^,^^46^ In brief, indicators of systemic inflammation were assessed with plasma IL-6 concentrations by ELISA, and microbial translocation was measured by plasma LPS concentrations with the Limulus Amebocyte Lysate assay.^39^ Frequencies of colon mDCs, pDCs, and T cells were determined using multi-color flow cytometry^39^^,^^40^ and expressed as an absolute number per gram of tissue based on the frequency within viable CD45+ cells, initial cell counts, and biopsy weights. Colon tissue HIV-1 RNA was quantified by real-time PCR and HIV-1 RNA copy numbers normalized per CD4 T cell within each biopsy calculated by the percent of all viable cells that were CD45+CD3+CD4+ as determined by flow cytometry and weight of each biopsy and reported as HIV-1 RNA per million CD4 T cell.^39^ Cytometric analyses of gut biopsies were performed on samples collected from uninfected controls and PLWH. Cytometric analyses that produced a low number of events due to technical issues were omitted from consideration for this study due to ensure accuracy. A full table of cytometric data used per sample is available at https://github.com/NicholasDopkins/JuneHIVMucosa/blob/main/Metadata.csv. The number of samples used per comparison can be found in the figure legends.

### Ethics

Analyses performed in this study were performed on deidentified and publicly available or previously archived data. The original clinical study was approved by the Colorado Multiple Institutional Review Board (COMIRB) and granted exempt research status. All study participants voluntarily provided written informed consent and the study was approved by the Colorado Multiple Institutional Review Board (COMIRB Protocol #11-0164).^15^^,^^16^^,^^39^^,^^40^

### Role of funders

The funding bodies had no influence on the planning, conduction and analysis of data performed during this study.

## Results

### HIV-1 status influences endogenous retroelement expression in the gut microenvironment

**Fig. 1:**
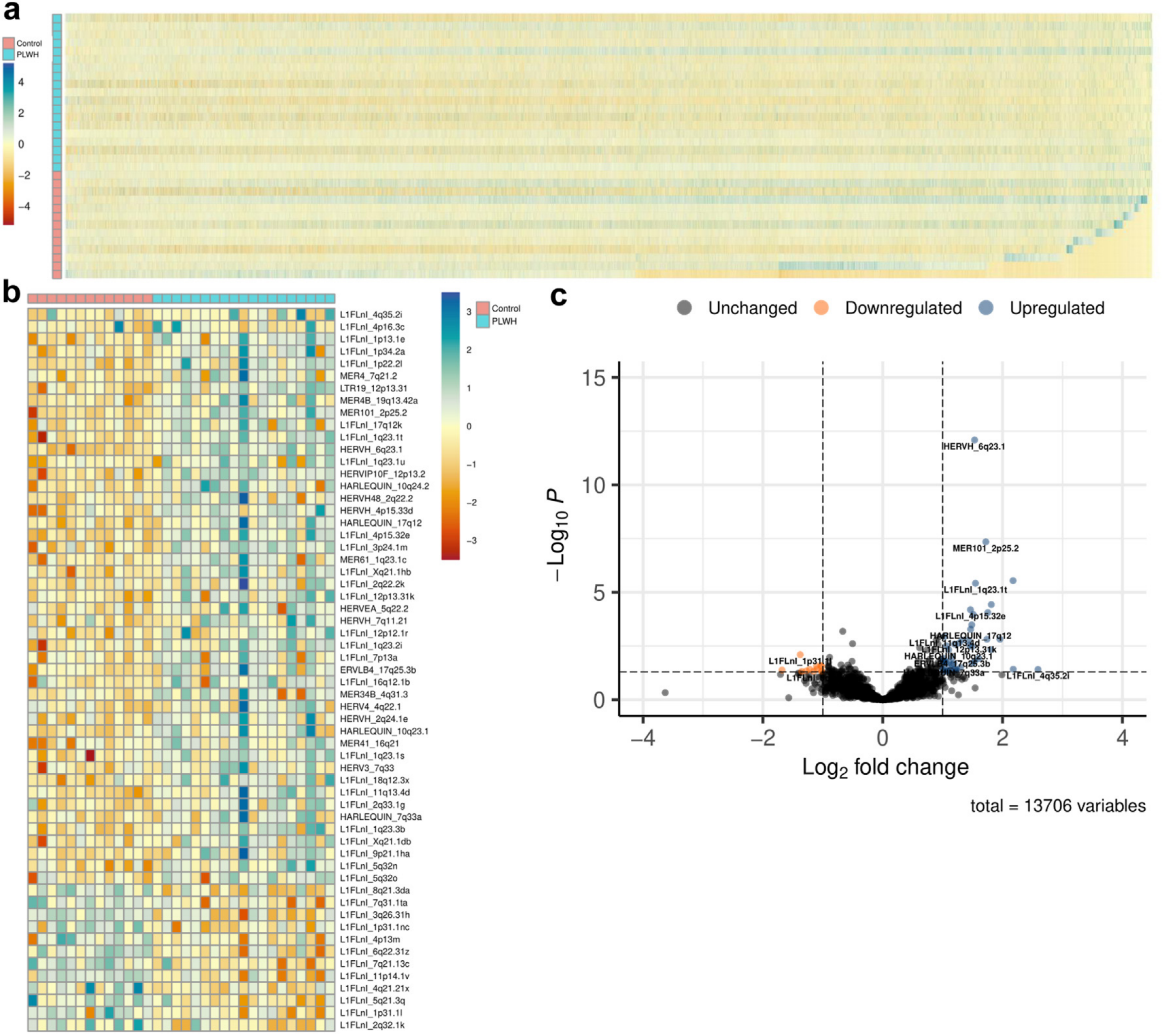
**HIV-1 status influences endogenous retroelement expression in the gut microenvironment**. Heatmap demonstrating the normalized per sample abundances of EREs following the preprocessing filtering of reads in uninfected controls vs PLWH. ERE reads were filtered to possess at least 2 reads in 10% of the total samples for quality assurance (a). Heatmap demonstrating the normalized per sample abundances differentially expressed EREs between uninfected control and PLWH gut biopsies. (b). Volcano plot demonstrating the average fold change and adjusted p value between uninfected control and PLWH gut biopsies. (c). [Uninfected controls (red) (n = 13) were compared against PLWH (blue) (n = 19); all statistics were performed in DESEQ using the Wald's Test; adjusted p values were calculated using default parameters for a Benjamini-Hochberg correction].

Following preprocessing to remove lowly abundant transcripts, we quantified the expression of 13,706 EREs in colon biopsies collected from PLWH in comparison to age and sex-matched HIV-1-uninfected controls (Fig. 1a). Differential expression was observed between controls and PLWH for 59 ERE loci (Fig. 1b and c). Of these 59 loci, 47 were upregulated and 12 were downregulated in PLWH. All HERVs differentially expressed were upregulated in PLWH when compared to controls. Overall, ERE activity at the RNA level displays discrete changes in the gut microenvironment in viremic individuals prior to the initiation of ART. Further consideration of the phylogenies and chromosomal origin of ERE expression profiles demonstrated no distinctive alterations in PLWH when compared to controls, suggesting no large-scale changes in ERE expression in the gut microenvironment of PLWH (Supplemental Fig. S1). To infer mechanisms by which HIV-1 infection induces the differential expression of EREs in the gut, we next quantified changes in expression resulting from IFN-I stimulation of uninfected gut-derived CD4+ T cells. IFN-Is do not shift the global landscape of ERE activity at the RNA level (Supplemental Fig. S2). Discrete changes in locus-specific expression demonstrate that IFN-I activation of uninfected gut CD4+ T cells share upregulated expression of 23 EREs, suggesting that they are IFN-inducible (Supplemental Figs. S3 and S4). 7 of these 23 EREs were upregulated in the gut of PLWH, suggesting immune-dependent mechanisms for their upregulation during HIV-1 infection, while 35 of the 42 upregulated EREs in PLWH showed no significant induction by IFN-I stimulation (Supplemental Fig. S5). Collectively, HIV-1 infection upregulates specific EREs in the gut of PLWH through both IFN-dependent and IFN-independent mechanisms. As diverse molecular interactions make HIV-1 and ERE activity sensitive to one another,^20^ further research is required to determine the exact cellular mechanisms responsible for the differential expression of EREs in the gut microenvironment of PLWH.

### Correlation between gut endogenous retroelement expression and viral load in people living with HIV-1

**Fig. 2:**
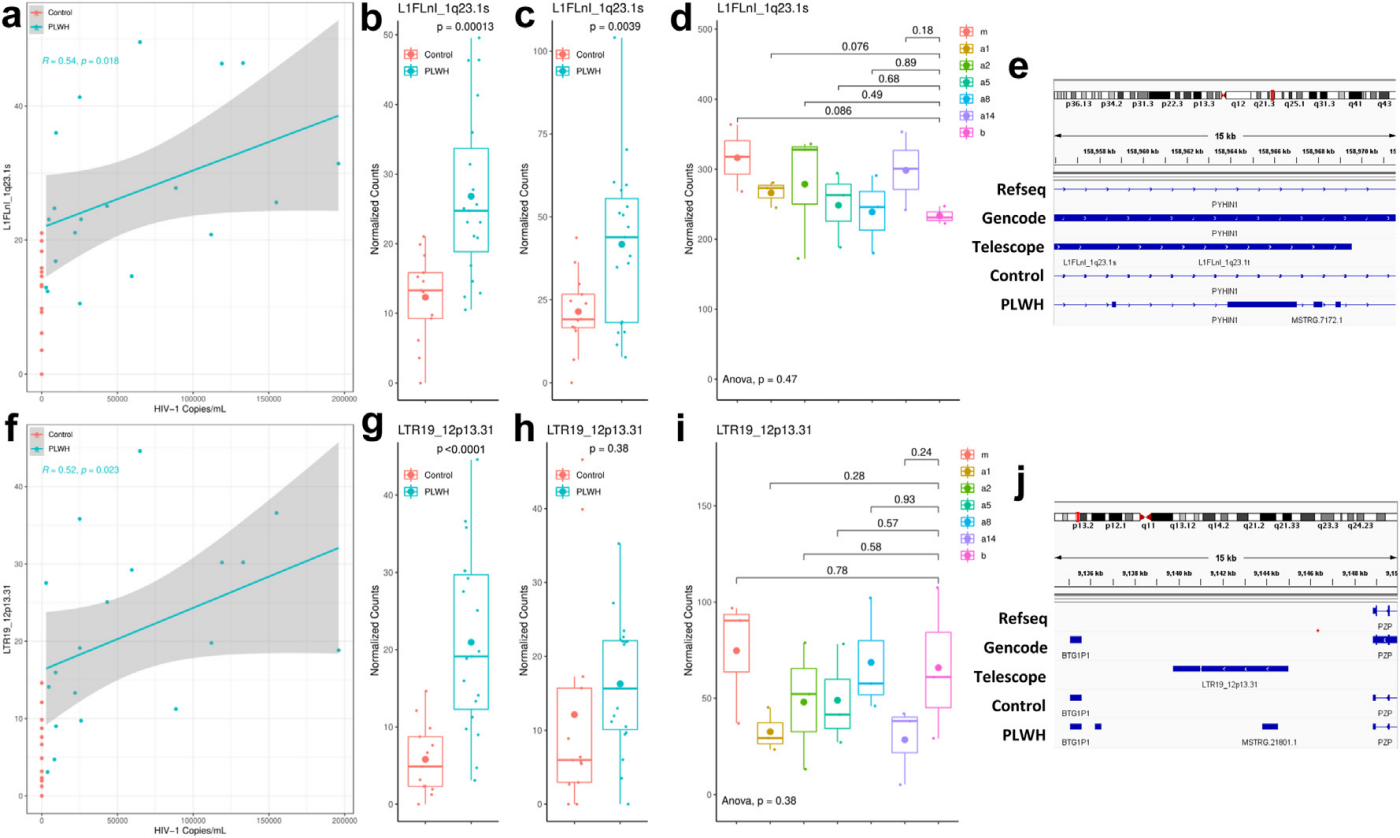
**Correlation between gut endogenous retroelement expression and viral load in people living with HIV-1**. Scatterplot demonstrating the viral load from plasma on the X axis and normalized expression of L1FlnI_1q23.1s in colon biopsies on the Y axis (a) [95% CI = 0.14–0.79, displayed p value was calculated using Spearman's correlation coefficient]. Normalized expression of L1FlnI_1q23.1s in colon biopsies from uninfected controls vs PLWH (b) [F = 0.27, num df = 12, denom df = 18, p-value = 0.027; displayed p value was generated using an unpaired t test]. Normalized expression of L1FLnI_1q23.1s in PBMCs from uninfected controls vs PLWH (c) [F = 0.22, num df = 12, denom df = 18, p-value = 0.011; displayed p value was generated using an unpaired t test]. Interferome array of L1FlnI_1q23.1s expression following IFN-a1, -a2, -a5, -a8, -a14, and -b treated gut CD4+ T cells when compared to mock (m) treatment (d) [Bartlett's K-squared = 6.90, df = 6, p-value = 0.33; One-way ANOVA performed for significance and padj values for individual comparisons were determined via Bonferroni corrected t testing for multiple comparisons]. IGV representation of L1FlnI_1q23.1s locus provided by the Telescope annotation alongside the Refseq and Gencode annotations. StringTie reconstructed transcriptomes demonstrate putative transcripts present in sequencing collected from the colon of uninfected controls and PLWH individuals (e). Scatterplot demonstrating the viral load from plasma on the X axis and normalized expression of LTR19_12p13.31 in colon biopsies on the Y axis (f) [95% CI = 0.14–0.82, displayed p value was calculated using Spearman's correlation coefficient]. Normalized expression of LTR19_12p13.31 in colon biopsies from uninfected controls vs PLWH (g) [F = 0.15, num df = 12, denom df = 18, F test p value = 0.0020; displayed p value was generated using an unpaired t test]. Normalized expression of LTR19_12p13.31 in PBMCs from uninfected controls vs PLWH (h) [F = 2.72, num df = 12, denom df = 18, p value = 0.053; displayed p value was generated using an unpaired t test]. Interferome array of LTR19_12p13.31 expression following IFN-a1, -a2, -a5, -a8, -a14, and -b treated gut CD4+ T cells when compared to mock (m) treatment (i) [Bartlett's K-squared = 2.64, df = 6, p value = 0.85; One-way ANOVA performed for significance and padj values for individual comparisons were determined via Bonferroni corrected t testing for multiple comparisons]. IGV representation of LTR19_12p13.31 locus provided by the Telescope annotation alongside the Refseq and Gencode annotations. StringTie reconstructed transcriptomes demonstrate putative transcripts present in sequencing collected from the colon of uninfected controls and PLWH individuals (j). [a1 (n = 3), a2 (n = 3), a5 (n = 3), a8 (n = 3), a14 (n = 3), and b (n = 3) were compared to m (n = 3); uninfected controls (red) (n = 13) were compared against PLWH (blue) (n = 19); shaded regions in scatterplots represent a 95% CI; Tukey box and whisker plots represent all replicates with small dots, median expression with a midline, and mean with an enlarged dot].

Spearman's correlation coefficients were calculated for all differentially expressed EREs in the gut of PLWH to identify any potential links with their expression and HIV-1 infection severity as determined by plasma HIV-1 viral load. Of the 59 differentially expressed EREs, four showed significant (p < 0.05) correlations between differential expression in the colon and plasma viral load in PLWH (L1FLnI_1q23.1s, LTR19_12p13.31, L1FlnI_4q21.21x and L1FlnI_3q26.31 h). L1FlnI_4q21.21x and L1FlnI_3q26.31 h were not considered for further analysis due to their deviation in expression being higher in uninfected controls and positively associating with viral load levels in PLWH (Supplemental Fig. S6), thus the regulation of their expression is likely resultant from physiological processes outside the scope of this study and their expression levels do not function as indicators of HIV-1 pathology. L1FlnI_1q23.1s possessed a positive association with viral load in the circulation (Fig. 2a) and is upregulated in the colon of PLWH when compared to uninfected controls (Fig. 2b). In PBMCs collected from the same participants, expression of L1FlnI_1q23.1s is upregulated in concurrence with gut expression of the element (Fig. 2c). Analysis of IFN-I activated gut CD4+ T cells suggests that no IFN-Is yielded any substantial effects on L1FlnI_1q23.1s (Fig. 2d). IGV demonstrates that the L1FlnI_1q23.1s locus is an ERE within the Pyrin and HIN Domain Family Member 1 (PYHIN1) gene with minor differences in the putative alignments provided by StringTie for uninfected controls and PLWH samples (Fig. 2e). LTR19_12p13.31 possessed a positive association with viral load in the circulation (Fig. 2f) and is upregulated in the colon of PLWH when compared to controls (Fig. 2g). In PBMCs collected from the same study participants, expression of LTR19_12p13.31 shows upregulation, however this was not statistically significant with the given sample size, therefore suggesting expression of the element in response to HIV-1 is weaker in systemic circulation when compared to the GI tract (Fig. 2h). Analysis of IFN-I activated gut CD4+ T cells suggests that IFN-Is yield no substantial effects on the expression of LTR19_12p13.31 (Fig. 2i). IGV demonstrates that the LTR19_12p13.31 locus is an intergenic ERE downstream of the 3′ UTR of Pregnancy Zone Protein (PZP) and produces a unique transcript in the putative alignments of PLWH samples provided by StringTie (Fig. 2j). Collectively, expression of the EREs L1FlnI_1q23.1s, and LTR19_12p13.31 show associations with blood HIV-1 RNA levels, and their induction in the gut may likely be independent of IFN-I mediated pathologies.

### Correlation between LTR19_12p13.31 and L1FlnI_1q23.1s expression and immune signatures of the gut microenvironment in people living with HIV-1

**Fig. 3:**
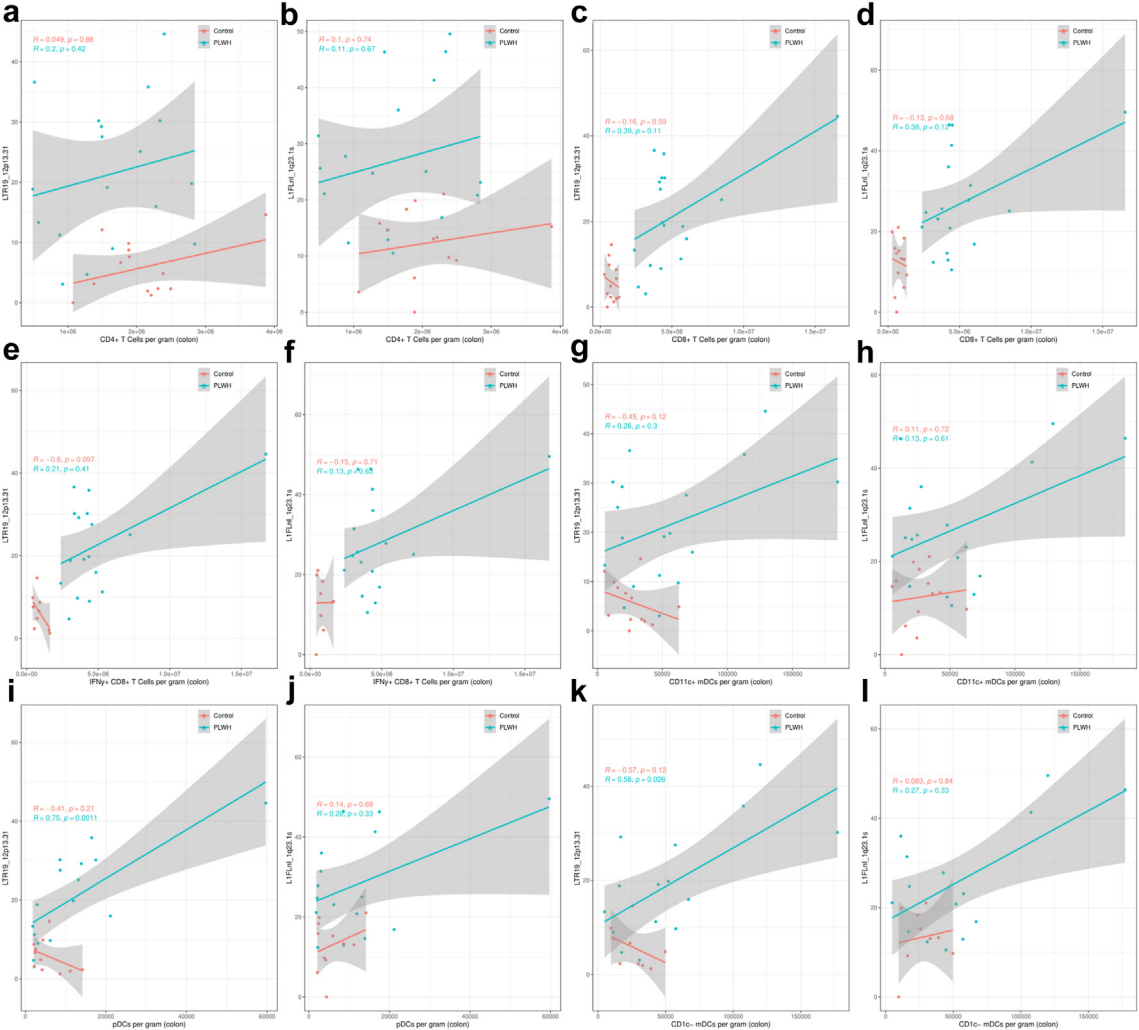
**Correlation between LTR19_12p13.31 and L1FLnI_1q23.1s expression and immune signatures of the gut microenvironment in people living with HIV-1**. Scatterplot demonstrating the CD4+ T Cells per gram on the X axis and normalized expression of LTR19_12p13.31 in colon biopsies on the Y axis (a) [Uninfected controls (red) (n = 13) 95% CI = −0.69 to 0.69, displayed p value was calculated using Spearman's correlation coefficient; PLWH (blue) (n = 19) 95% CI = −0.31 to 0.65, displayed p value was calculated using Spearman's correlation coefficient]. Scatterplot demonstrating the CD4+ T Cells per gram on the X axis and normalized expression of L1FLnI_1q23.1s in colon biopsies on the Y axis (b) [Uninfected controls (red) (n = 13) 95% CI = −0.52 to 0.60; displayed p value was calculated using Spearman's correlation coefficient; PLWH (blue) (n = 19) 95% CI = −0.3 to 0.56; displayed p value was calculated using Spearman's correlation coefficient]. Scatterplot demonstrating the CD8+ T Cells per gram on the X axis and normalized expression of LTR19_12p13.31 in colon biopsies on the Y axis (c) [Uninfected controls (red) (n = 13) 95% CI = −0.68 to 0.45; displayed p value was calculated using Spearman's correlation coefficient; PLWH (blue) (n = 19) 95% CI = −0.13 to 0.74; displayed p value was calculated using Spearman's correlation coefficient]. Scatterplot demonstrating the CD8+ T Cells per gram on the X axis and normalized expression of L1FLnI_1q23.1s in colon biopsies on the Y axis (d) [Uninfected controls (red) (n = 13) 95% CI = −0.75 to 0.51; displayed p value was calculated using Spearman's correlation coefficient; PLWH (blue) (n = 19) 95% CI = −0.07 to 0.71; displayed p value was calculated using Spearman's correlation coefficient]. Scatterplot demonstrating the IFNy + CD8+ T Cells per gram on the X axis and normalized expression of LTR19_12p13.31 in colon biopsies on the Y axis (e) [Uninfected controls (red) (n = 9) 95% CI = −0.98 to 0.22; displayed p value was calculated using Spearman's correlation coefficient; PLWH (blue) (n = 18) 95% CI = −0.38 to 0.70; displayed p value was calculated using Spearman's correlation coefficient]. Scatterplot demonstrating the IFNy + CD8+ T Cells per gram on the X axis and normalized expression of L1FLnI_1q23.1s in colon biopsies on the Y axis (f) [Uninfected controls (red) (n = 9) 95% CI = −0.84 to 0.62; displayed p value was calculated using Spearman's correlation coefficient; PLWH (blue) (n = 18) 95% CI = −0.36 to 0.54; displayed p value was calculated using Spearman's correlation coefficient]. Scatterplot demonstrating the CD11c + mDCs per gram on the X axis and normalized expression of LTR19_12p13.31 in colon biopsies on the Y axis (g) [Uninfected controls (red) (n = 13) 95% CI = −0.84 to 0.07; displayed p value was calculated using Spearman's correlation coefficient; PLWH (blue) (n = 19) 95% CI = −0.24 to 0.65; displayed p value was calculated using Spearman's correlation coefficient]. Scatterplot demonstrating the CD11c + mDCs per gram on the X axis and normalized expression of L1FLnI_1q23.1s in colon biopsies on the Y axis (h) [Uninfected controls (red) (n = 13) 5% CI = −0.48 to 0.70; displayed p value was calculated using Spearman's correlation coefficient; PLWH (blue) (n = 19) 844; 95% CI = −0.43 to 0.64; displayed p value was calculated using Spearman's correlation coefficient]. Scatterplot demonstrating the pDCs per gram on the X axis and normalized expression of LTR19_12p13.31 in colon biopsies on the Y axis (i) [Uninfected controls (red) (n = 11) 95% CI = −0.84 to 0.38; displayed p value was calculated using Spearman's correlation coefficient; PLWH (blue) (n = 17) 95% CI = 0.37–0.93; displayed p value was calculated using Spearman's correlation coefficient]. Scatterplot demonstrating the pDCs per gram on the X axis and normalized expression of L1FLnI_1q23.1s in colon biopsies on the Y axis (j) [Uninfected controls (red) (n = 11) 95% CI = −0.61 to 0.77; displayed p value was calculated using Spearman's correlation coefficient; PLWH (blue) (n = 17) 95% CI = −0.35 to 0.75; displayed p value was calculated using Spearman's correlation coefficient]. Scatterplot demonstrating the CD1c-mDCs per gram on the X axis and normalized expression of LTR19_12p13.31 in colon biopsies on the Y axis (k) [Uninfected controls (red) (n = 9) 95% CI = −1.00 to 0.11; displayed p value was calculated using Spearman's correlation coefficient; PLWH (blue) (n = 16) 95% CI = −0.08 to 0.87; displayed p value was calculated using Spearman's correlation coefficient]. Scatterplot demonstrating the CD1c-mDCs per gram on the X axis and normalized expression of L1FLnI_1q23.1s in colon biopsies on the Y axis (l) [Uninfected controls (red) (n = 9) 95% CI = −0.88 to 0.83; displayed p value was calculated using Spearman's correlation coefficient; PLWH (blue) (n = 16) 95% CI = −0.44 to 0.75; displayed p value was calculated using Spearman's correlation coefficient]. [Shaded regions in scatterplots represent a 95% CI].

We next analysed how expression of L1FlnI_1q23.1s and LTR19_12p13.31 correlate with cellular abundances that may be associated with pathogenesis in the gut of PLWH. There were no significant associations between the number colon CD4+ T cells (measured per gram of gut tissue) with LTR19_12p13.31 (Fig. 3a) and L1FlnI_1q23.1s expression (Fig. 3b). LTR19_12p13.31 and L1FlnI_1q23.1s demonstrated positive correlations with the number of colonic CD8+ T cells and IFN gamma (IFNy) + CD8+ T cells (Fig. 3c–f). These associations between immune cell subsets and ERE activity were not statistically significant given the sample sizes, however a clear distinction between the number of IFNy + CD8+ T cells between groups and the expression of LTR19_12p13.31 and L1FlnI_1q23.1s can be observed, suggesting further study. Further consideration into dendritic cell (DC) abundances demonstrated that LTR19_12p13.31 (Fig. 3g) and L1FlnI_1q23.1s (Fig. 3h) show weak positive correlations with CD11c + myeloid DCs (mDCs). The ERE LTR19_12p13.31 (Fig. 3i) displayed a significant positive correlation and L1FlnI_1q23.1s (Fig. 3j) displayed an insignificant positive association with plasmacytoid DCs (pDCs). Amongst mDCs, LTR19_12p13.31 (Fig. 3k) showed s significant positive correlation with the CD1c-mDCs and L1FlnI_1q23.1s (Fig. 3l) showed an insignificant positive correlation with CD1c-mDCs, but neither showed any association with the abundances of CD1c + mDCs (Supplemental Fig. S7), suggesting potential perturbations related to specific mDC subsets.^40^ In analyses of frequencies of CD4 T helper (Th) cell subsets, L1FlnI_1q23.1s significantly positively correlated with IFNy-IL17+ CD4+ T cells (i.e., Th17) and with IFNy + IL17+ CD4+ T cells (i.e., inflammatory Th17 cells) (Supplemental Fig. S8). LTR19_12p13.31 (Supplemental Fig. S9) did not demonstrate significant correlations with analysed CD4+ T cell subset abundances in the gut. Furthermore, systemic markers of microbial translocation and inflammation, including plasma lipopolysaccharide (LPS) and interleukin (IL)-6, demonstrated no attributable associations with LTR19_12p13.31 or L1FlnI_1q23.1s expression (Supplemental Fig. S10). Collectively, LTR19_12p13.31 and L1FlnI_1q23.1s may be associated with certain immune cell frequencies observed in the gut microenvironment of PLWH. In this study, multiple correlations possess considerable 95% CI ranges that span both negative and positive R values despite significant p values, likely being driven in part by the limited size of the described cohort being further cofounded by individual heterogeneity. Despite this limitation, a strong positive association is observed between pDC abundances associated with HIV-1 gut pathology and LTR19_12p13.31 expression in PLWH (95% CI = 0.37–0.93), suggesting the importance of future studies on ERE activity in the gut of PLWH.

## Discussion

In this study, we leverage modulations in ERE activity at the RNA level in the GI tract of untreated PLWH with frequencies of immune cell populations that are likely critical players in driving HIV-1 gut pathogenesis.^9^ As the derepression of EREs may possess immunomodulatory roles in human diseases,^27^, ^28^, ^29^ our results suggest further investigation into their activity in HIV-1 pathogenesis. Analysis of RNA sequencing data from colon biopsies of uninfected controls vs untreated PLWH identified 59 differentially expressed EREs associated with HIV. By finding correlations between their colonic expression and circulating viral load, we were able to focus on 2 EREs upregulated in PLWH that might indicate the severity of HIV-1 pathogenesis in the gut, or function as markers of immune deregulation. The EREs LTR19_12p13.31 and L1FlnI_1q23.1s best correlated with cellular abundances including higher frequencies of pDCs, CTLs, and CD1c-mDCs. By considering their expression in PBMCs from the same study participants, we observed a significant upregulation in L1FlnI_1q23.1s expression and a non-significant upregulation in LTR19_12p13.31. In accordance with previous studies,^47^ we observe significant alterations in ERE activity at the RNA level in the PBMCs of PLWH and uninfected controls (Supplemental Fig. S11). This suggests future use of L1FlnI_1q23.1s as a potential biomarker for deregulations of immunity in the gut microenvironment of PLWH, while LTR19_12p13.31 is more likely a biomarker specific to the GI tract. Collectively, we find significant associations between LTR19_12p13.31 and L1FlnI_1q23.1s and cellular dynamics of the GALT in PLWH, further emphasizing their identity as biomarkers or potential mediators of immune deregulation.

Previous studies have demonstrated that ERE activity is widely changed in response to the HIV-1 lifecycle^20^^,^^21^ and certain HIV-1 associated comorbidities.^48^^,^^49^ ERE expression is also susceptible to changes the in epigenetics and immunity that are elicited during viral infections and the host response.^50^ While it remains unknown if the changes in expression for most EREs result from a bystander effect or by a cooption of host immunity in HIV-1 infection, their activity can be incorporated to better characterize poorly understood immunopathologies in viral infections. Here, we demonstrate that significant correlations can be established between differentially expressed EREs and cellular markers that likely contribute to GI immunopathologies of untreated HIV-1 infection.

The GI tract is a common HIV-1 reservoir site that poses unique obstacles in the eradication of integrated proviruses and containment of proviral transcription. The barriers of the lower GI tract are laden with host-microbe immune interactions that condition the host immune response and influence systemic immunity. This anatomically unique and physiologically complex network of immune conditioning is sensitive to external cues from infections and fluctuations in the composition of GI microorganisms. In PLWH, the GI microenvironment can permit the rapid infectivity of HIV-1 to local CD4+ T cells, and therefore lead to their depletion.^51^^,^^52^ Disruption of GALT immune dynamics in PLWH is common, and HIV-1 pathogenesis is likely worsened by the sustainment of elevated LPS^53^ and IL-6^52^ as a consequence of the alteration in gut homeostasis. In this cohort it was previously shown that PLWH display elevated LPS and IL-6 in their plasma contents,^15^ however these elevations do not correlate with LTR19_12p13.31 and L1FlnI_1q23.1s activity. We therefore interpret that increased levels of circulating LPS and IL-6 do function as a chronic consequence of HIV-1 infection that reinforces pathogenesis independently from LTR19_12p13.31 and L1FlnI_1q23.1s activity, however this observation may be limited by the size of our cohort and requires further study.

We note some limitations of the study. First, we were unable to discern what cell types are responsible for the overexpression of differentially expressed EREs, as the study utilized bulk RNA sequencing data. As the cellular composition of the sequenced tissues fluctuates with disease severity, it is unclear whether the phenotype or mere abundance of disproportionate cell types, such as pDCs and CD8 T cells, is responsible for their expression. In applying interferome analyses of uninfected CD4+ T cells, we hoped to elucidate a mechanism driving ERE deregulation in the GI tract of PLWH. It is possible that IFN-I stimulation of other cell types in the GI tract or of HIV-1 infected gut CD4+ T cells may be responsible for changes in ERE activity at the RNA level. This study performed a retrospective analysis of previously acquired datasets collected as part of a prior clinical study. Although this provided a unique opportunity to investigate retroelement expression in gut tissue of people with and without HIV, power calculations were not performed, and analyses may be limited by small samples sizes. Cohorts were matched to account for age and sex as described in Supplemental Table S1, however additional confounding factors may (e.g., dietary and smoking habits). Future studies that accurately quantify ERE expression from single cell RNA sequencing data would provide further insight into the activation state of these EREs in the GI tract of PLWH. Analysis of ERE activity in single cells at mucosal barriers in PLWH may likely define how lowly abundant cell types participate in HIV-1 driven immunopathologies as well as describe markers of infected cells that could aid in eradication of viral reservoirs. Additionally, EREs are repetitive and may be difficult to accurately assign to genome annotations in short read sequencing data.^54^ To address this issue, we utilized the bioinformatic pipeline “Telescope” to quantify their expression, which more accurately reassigns ambiguous ERE through the application of an expectation maximization (EM)-based algorithm. While EM-based approaches such as Telescope can improve upon the classification of ambiguous reads from short read RNA sequencing data, future implementation of long read RNA sequencing for ERE quantification in the gut of PLWH may likely increase definition and identify additional biomarkers not observed in this study. Additionally, due to the poorly defined molecular characteristics and low copy number of LTR19 HERV sequences, exceedingly little is known about their potential roles in health and disease. LTR19 sequences are a member of the HERVFA clade and are estimated to only possess roughly ∼15 copies in the human genome.^55^

In conclusion, this study provides a correlative link between ERE expression and the deregulations of GALT during untreated chronic HIV-1 infection. While EREs can be modulated in context dependent mechanisms through various aspects of HIV-1 infection, their potential roles in gut mucosal immunity have yet to be defined. We also demonstrate that the IFN-I response mediates ERE expression in uninfected gut CD4+ T cells, however this only appears to be responsible for a distinct subset of differentially expressed elements among PLWH and do not include LTR19_12p13.31 or L1FlnI_1q23.1s. We find that LTR19_12p13.31 and L1FLnI_1q23.1s are promising biomarkers of HIV-1 pathogenesis in the gut and highlight a differentially expressed LTR19 element in a human disease, thus emphasizing its importance for further study.

## Contributors

ND co-designed the study, co-prepared the first manuscript draft, co-performed data analyses, co-curated code, led statistical analyses, co-verified the underlying data, and curated figures. TF co-prepared the first manuscript draft, co-performed data analyses, co-performed statistical analysis, co-curated code, co-verified the underlying data, and provided intellectual input critical to analysis. SM co-designed the study, co-prepared the first manuscript draft, co-performed data analyses, and provided intellectual input critical to data interpretation. NL co-performed analyses and co-curated code. KG provided intellectual input critical to hypothesis generation and co-provided data access. KLM provided intellectual input critical to hypothesis generation and co-provided data access. BSB provided intellectual input critical to hypothesis generation and co-provided data access. MLB provided intellectual input critical to hypothesis testing. SMD provided intellectual input critical to hypothesis generation and testing, co-provided data access, and performed immunological analysis of the gut microenvironment. CCW provided intellectual input critical to hypothesis generation and co-provided data access. DFN co-designed the study and co-prepared the first manuscript draft. MLS co-designed the study, co-prepared the first manuscript draft, and co-provided data access. All authors contributed to data interpretation, discussion regarding significances, and editing of the manuscript. All authors have read and approved the final manuscript.

## Data sharing statement

FASTQ files from the bulk RNA sequencing performed on colon biopsies reanalysed by this study can be accessed at the NCBI sequence read archive (SRA) under PRJNA558500. FASTQ files from the bulk RNA sequencing performed on healthy gut CD4+ T cells treated with IFN-Is reanalysed by this study can be accessed at the NCBI SRA under PRJNA558974. FASTQ files from the bulk RNA sequencing performed on PBMCs reanalysed by this study are available upon request and to be released to the NCBI SRA under PRJNA659373. For raw file data access prior to release, the data is available upon request by contacting kejun.guo@cuanschutz.edu. A preprocessed read table of the Telescope outputs for PBMC data analysed by this study is available at https://github.com/NicholasDopkins/JuneHIVMucosa/blob/main/PBMC_Telescope_Output.csv. Code for analysis can be accessed at https://github.com/NicholasDopkins/JuneHIVMucosa. Details on running the Telescope pipeline can be accessed at https://github.com/mlbendall/telescope.

## Declaration of interests

All authors have no potential conflicts of interests to disclose.
