# Supplementary material for: Endogenous retroelement expression in the gut microenvironment of people living with HIV-1
Source: eBioMedicine. 2024 Apr 26;103:105133. doi: 10.1016/j.ebiom.2024.105133 (PMC11061259; doi:10.1016/j.ebiom.2024.105133)
Supplement: Supplemental Table S1 [file mmc1.docx]

**Supplemental Table**

|  | Uninfected controls | People living with HIV-1 (PLWH) | P value |
| --- | --- | --- | --- |
| Number of study participants | 13 | 19 |  |
| Age |  |  |  |
| *Median (IQR) (years)* | 33 (27.5-45.0) | 33 (26.0-46.0) | 0.96 |
| Sex |  |  |  |
| *Male* | 8 | 13 | 0.72 |
| *Female* | 5 | 6 |  |
| Ethnicity |  |  |  |
| *Non-Hispanic* | 11 | 14 | 0.67 |
| *Hispanic* | 2 | 5 |  |
| Race |  |  |  |
| *White/Caucasian* | 9 | 15 | 0.62 |
| *Black/African American* | 2 | 3 |  |
| *Asian* | 2 | 1 |  |
| Men who have sex with men (MSM) |  |  |  |
| *Yes* | 1 | 12 | 0.0028 |
| *No* | 12 | 7 |  |
| Reported intravenous drug use (IDU)* |  |  |  |
| *Yes* | 0 | 4 | 0.11 |
| *No* | 14 | 14 |  |
| Blood CD4 count |  |  |  |
| *Median (IQR) (cells/μl)* | 720 (560-1006) | 429 (340-624) | 0.0015 |
| Plasma HIV viral load |  |  |  |
| *Median (IQR) (RNA copies/ml)* | N/A | 26000 (9180-112000) |  |
| Years since first HIV-1 seropositive test |  |  |  |
| *Median (IQR)* | N/A | 3.5 (1.5-10) |  |
