# Supplementary material for: Endogenous retroelement expression in the gut microenvironment of people living with HIV-1
Source: eBioMedicine. 2024 Apr 26;103:105133. doi: 10.1016/j.ebiom.2024.105133 (PMC11061259; doi:10.1016/j.ebiom.2024.105133)
Supplement: Supplemental Figs S1–S11 [file mmc2.pptx]

## Slide 1
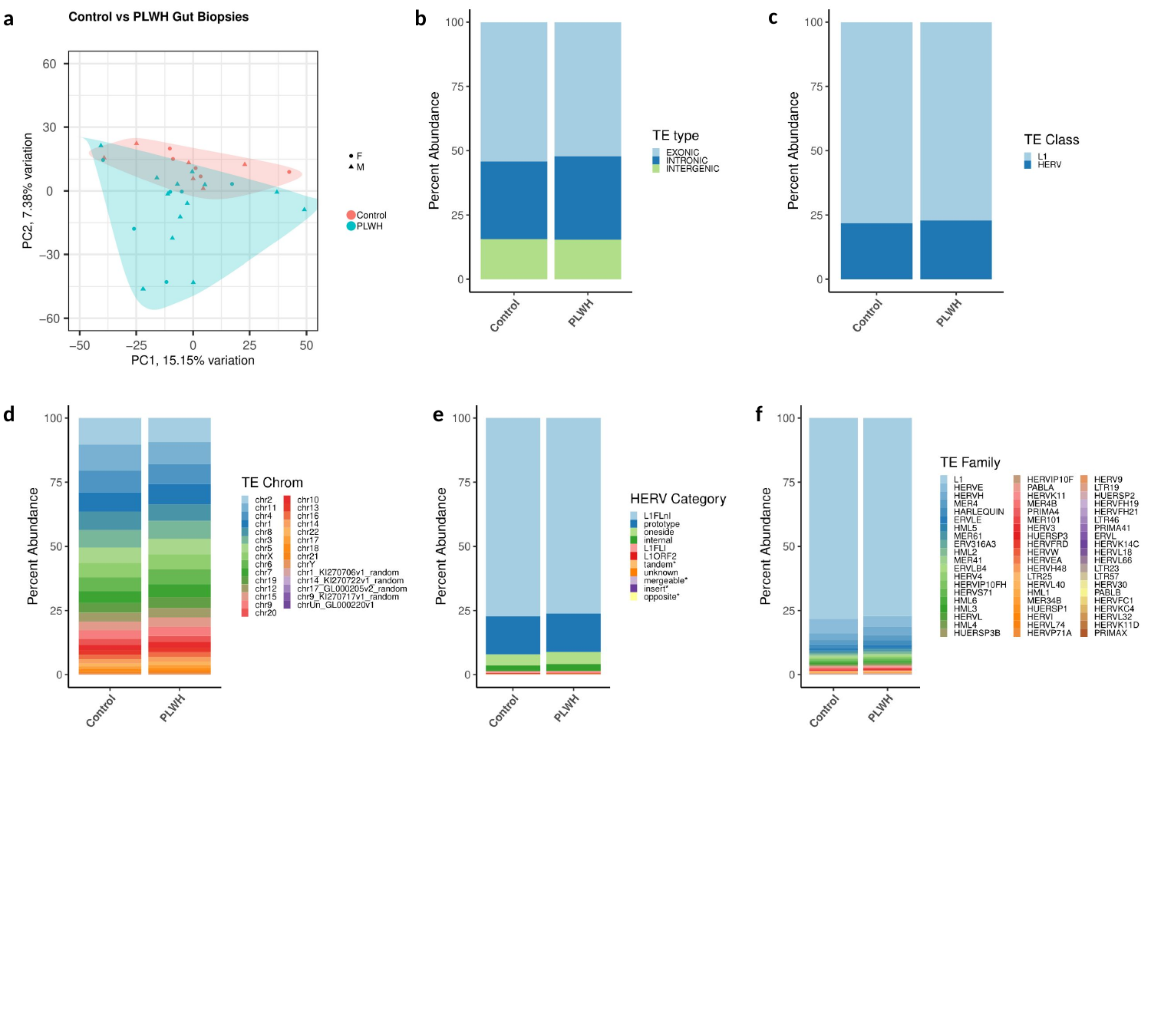

c
a
b
d
e
f

## Slide 2
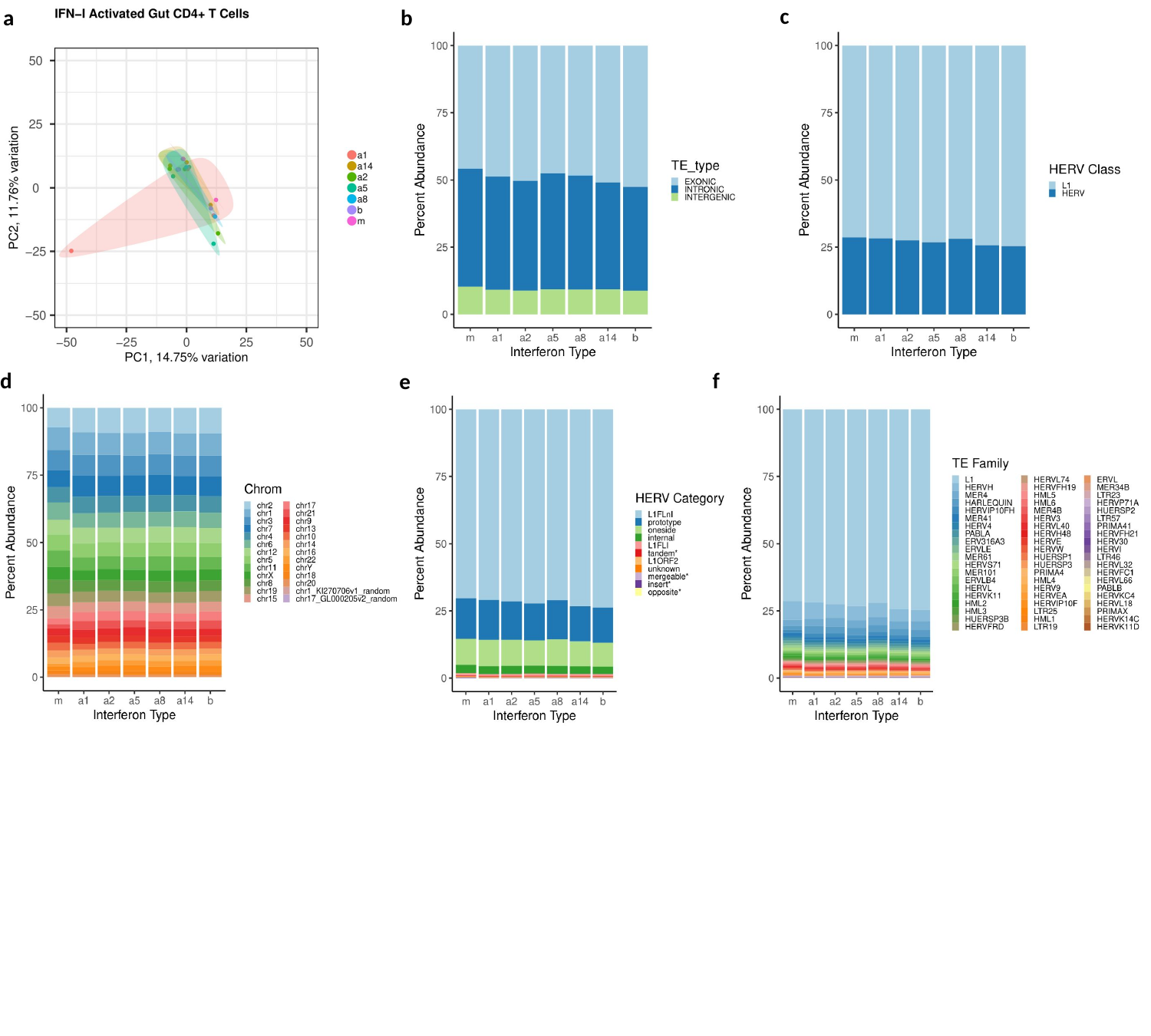

c
a
b
d
f
e

## Slide 3
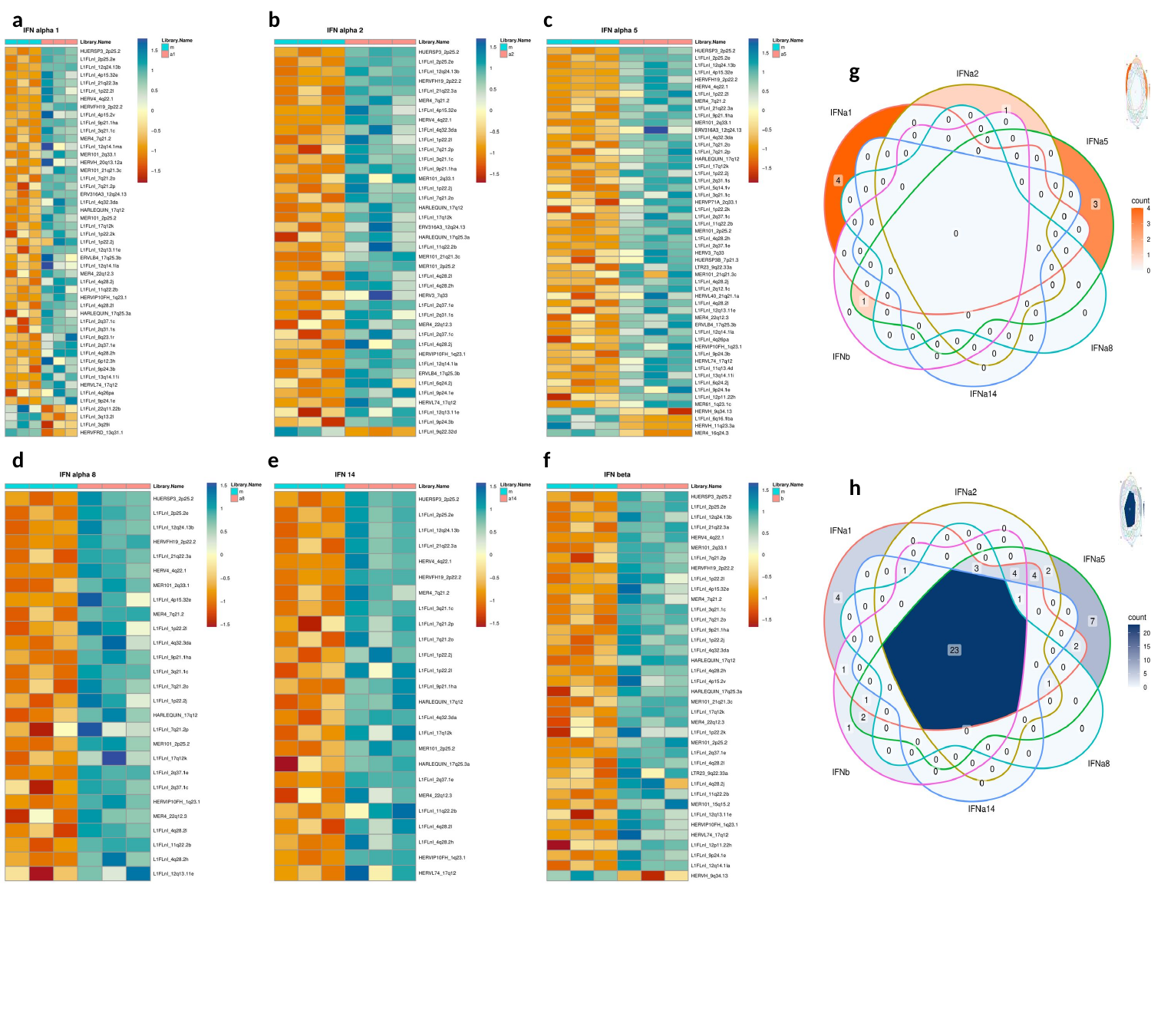

a
b
c
g
d
e
f
h

## Slide 4
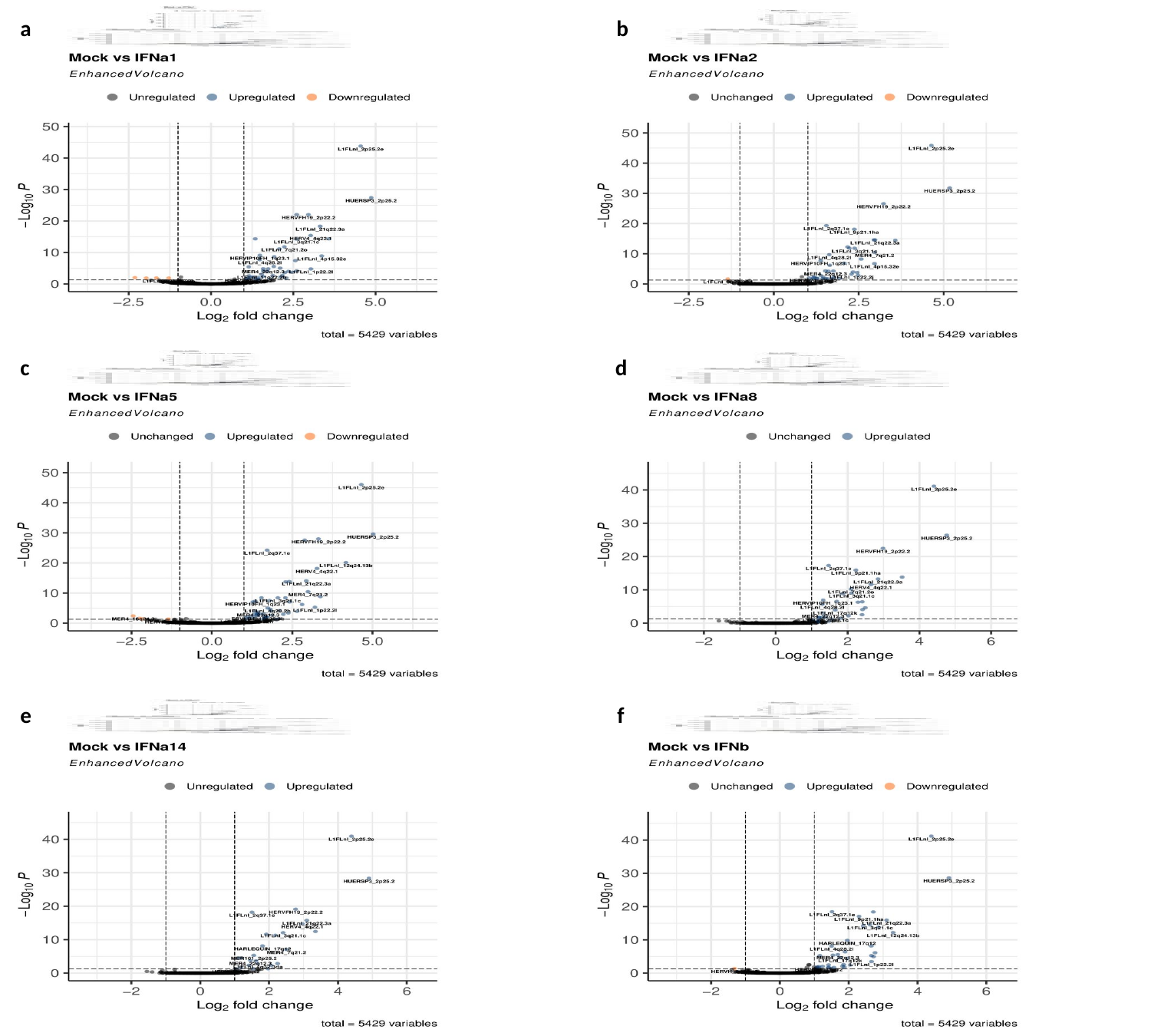

a
b
c
d
e
f

## Slide 5
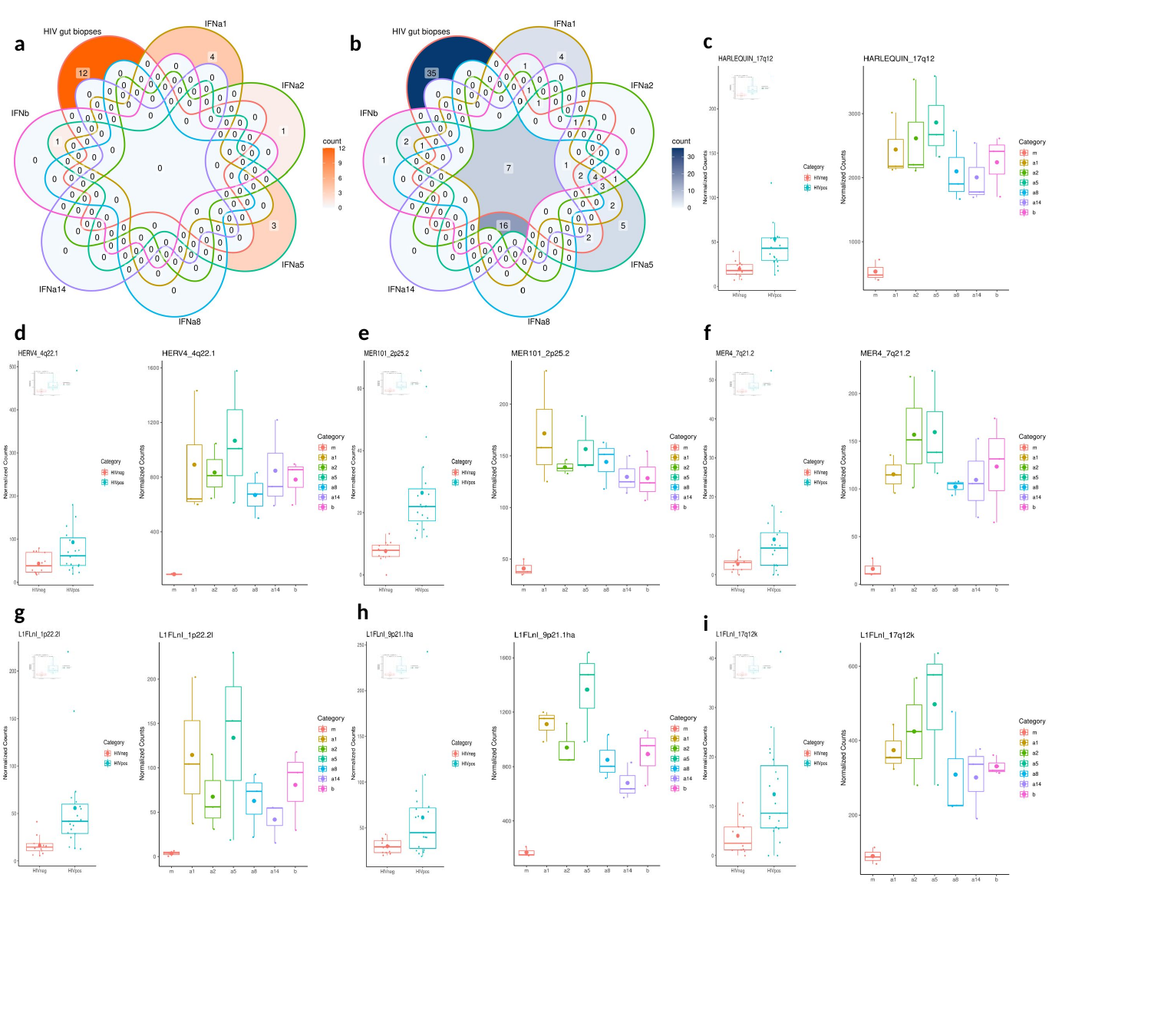

c
a
b
d
e
f
g
h
i

## Slide 6
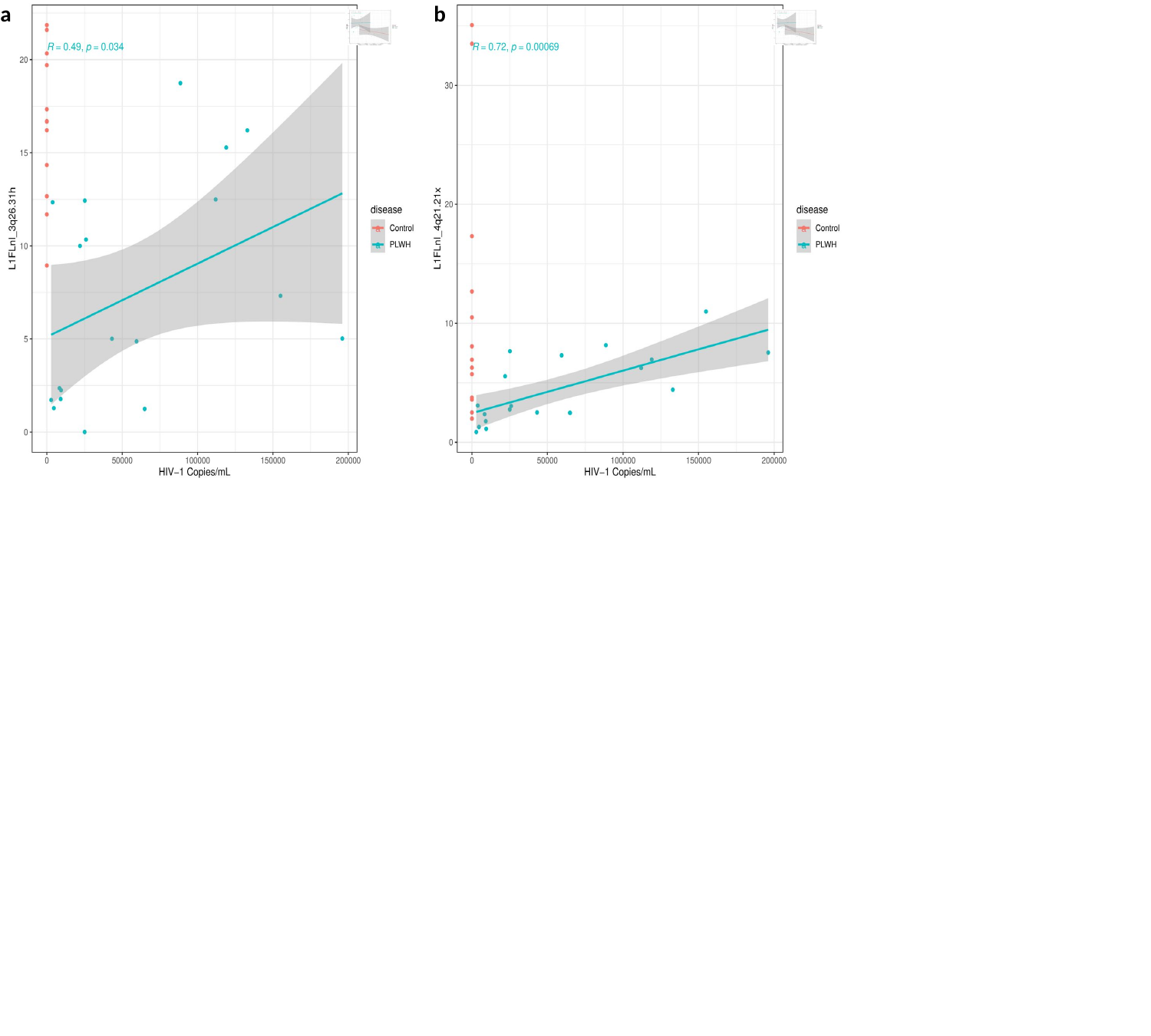

a
b

## Slide 7
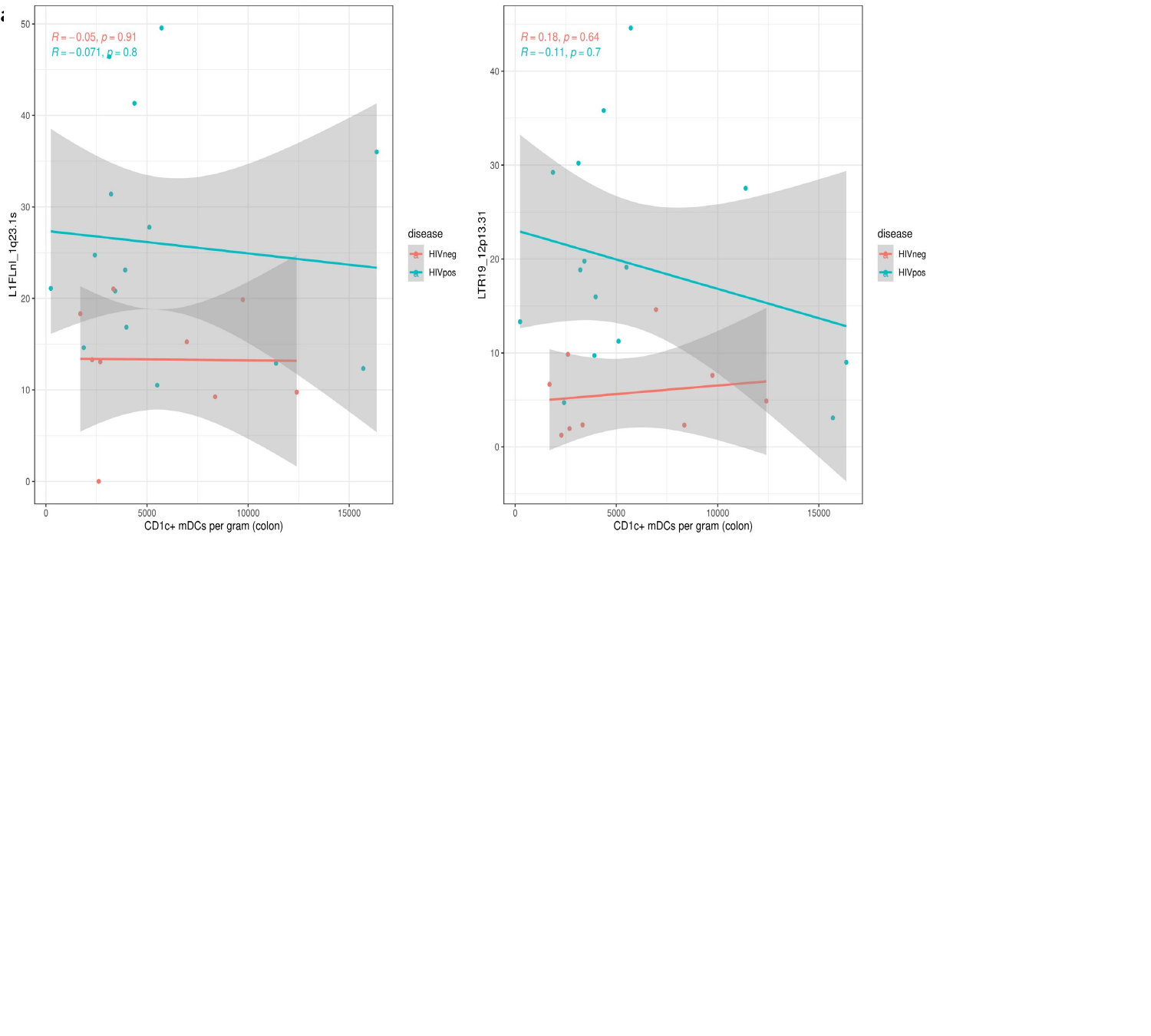

a
b

## Slide 8
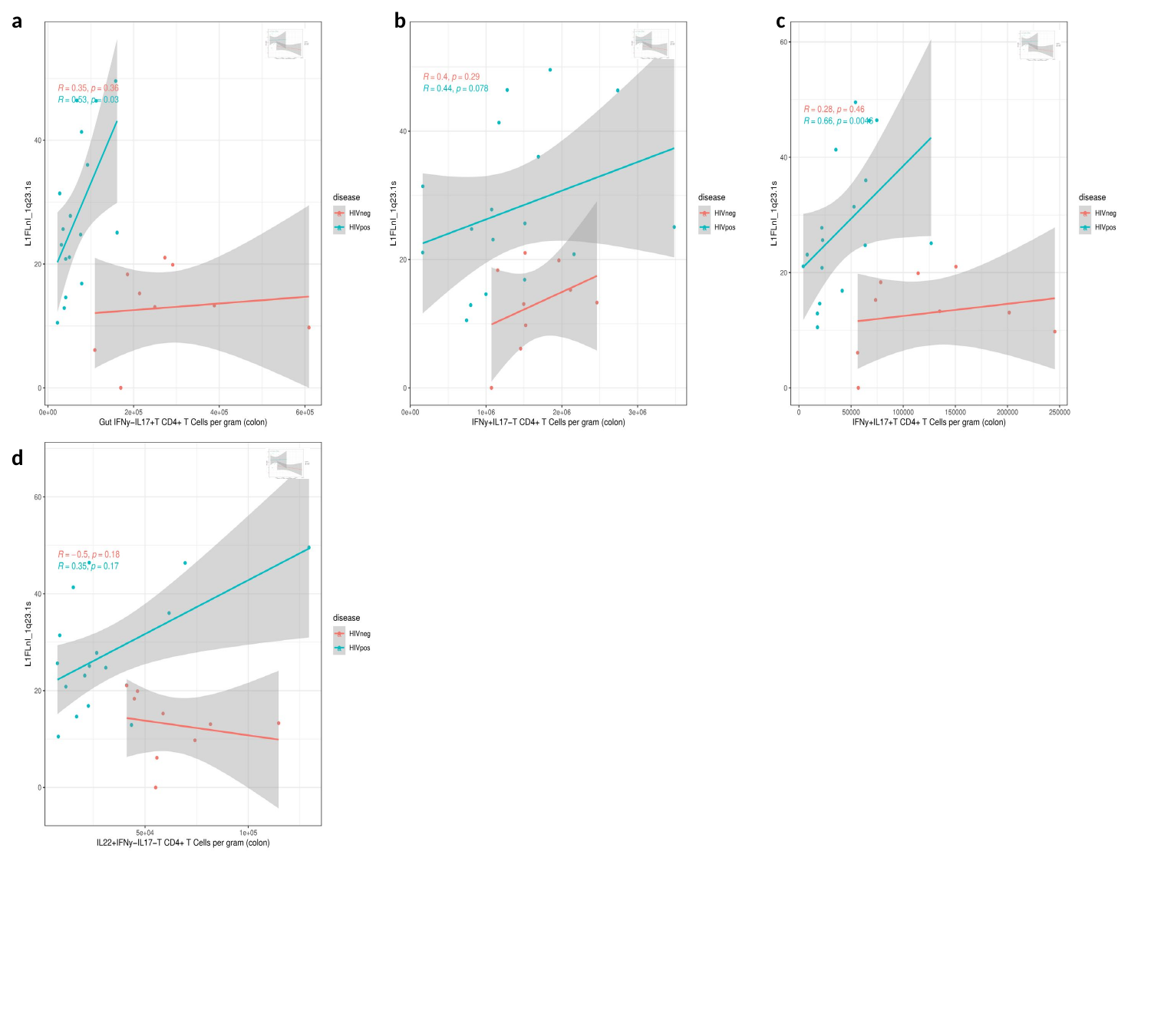

a
b
c
d

## Slide 9
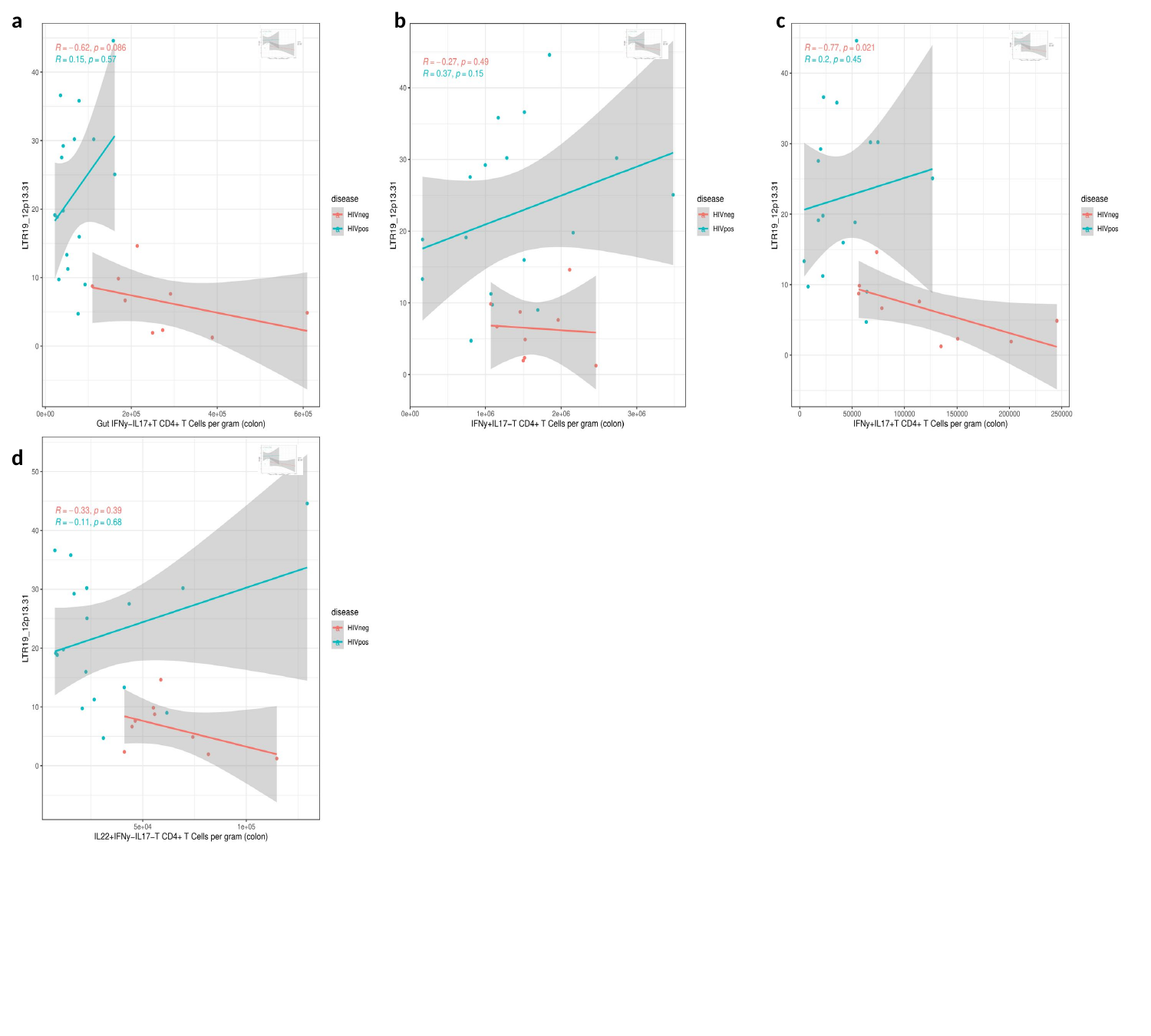

a
b
c
d

## Slide 10
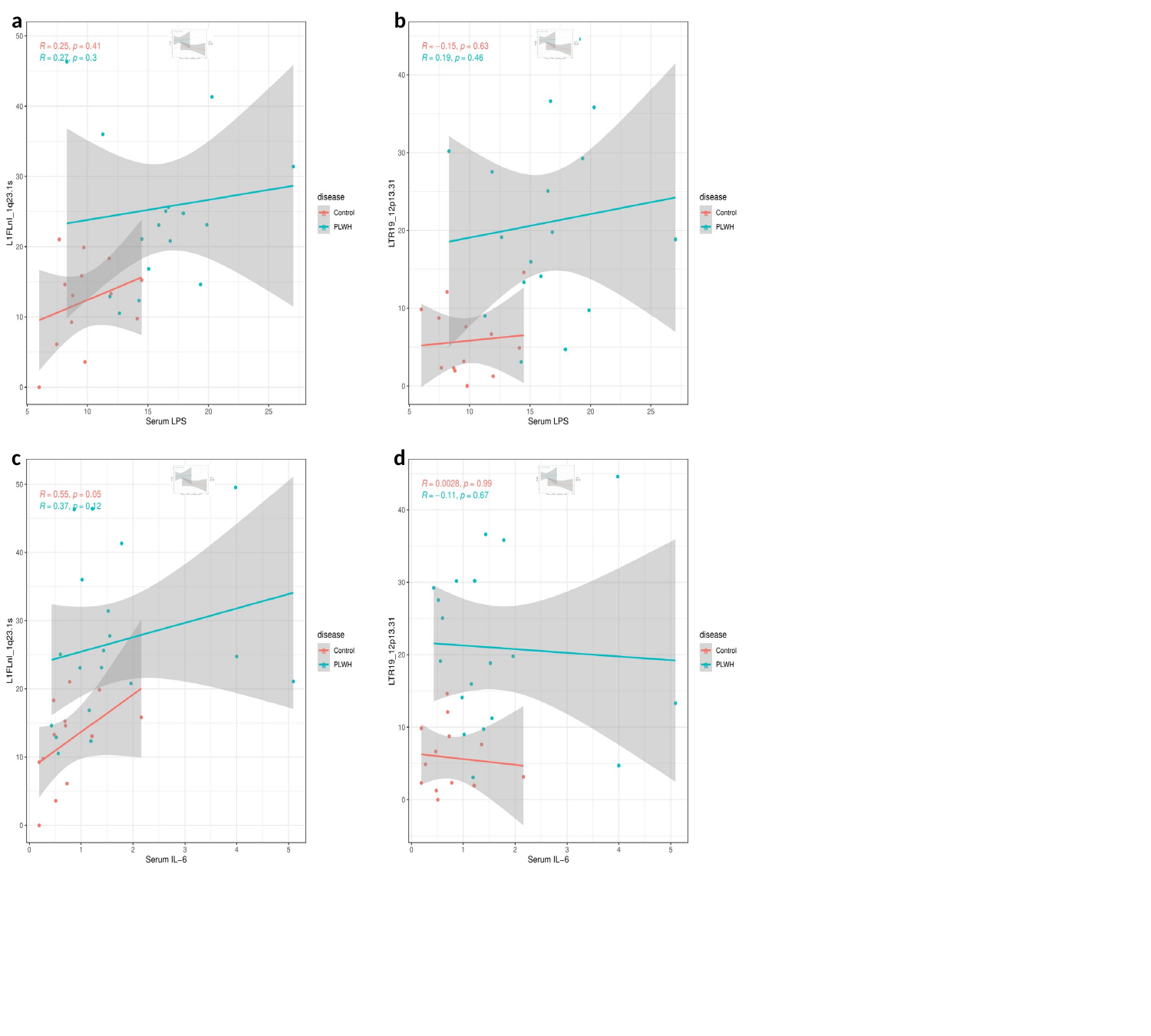

a
b
c
d

## Slide 11
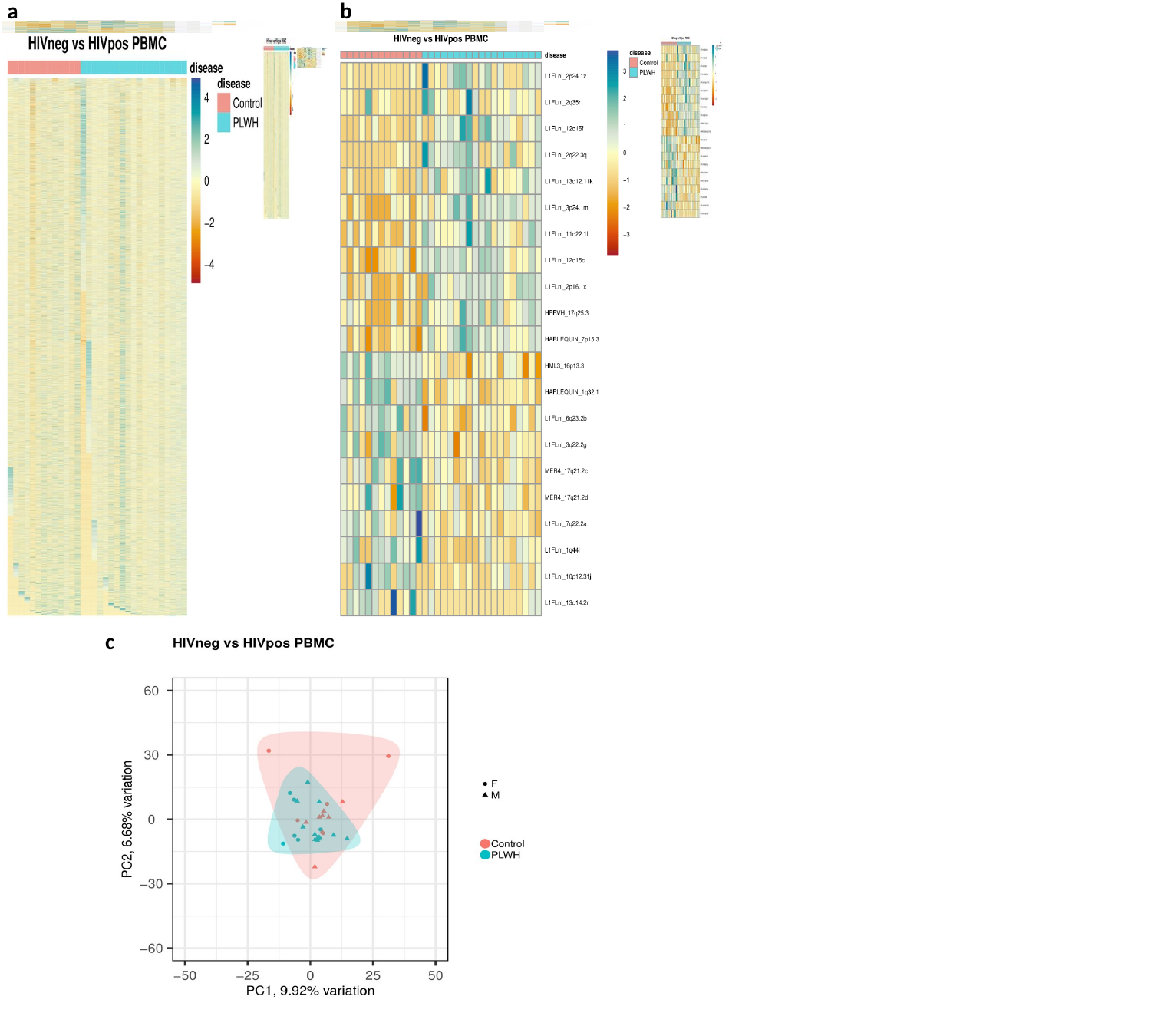

a
b
c
